# Diagnostic challenges in the older patient

**DOI:** 10.1186/2045-709X-20-28

**Published:** 2012-09-05

**Authors:** Lisa Zaynab Killinger

**Affiliations:** 1 Director of Diagnosis and Radiology, Palmer College of Chiropractic, 1000 Brady Street, 52803, Davenport, IA, USA

**Keywords:** Diagnosis, Geriatrics, Polypharmacy, Cognitive health, Delirium, Dementia, Depression, Pathologies, Chiropractic

## Abstract

Older patients often present with a long, complex history and a clinical picture that frequently includes co-morbidities. It is essential that health professionals caring for older patients become familiar with common age-related changes, and the specific clinical factors that complicate the diagnostic process. A case-based approach is taken in this article to explore the diagnostic challenges in caring for older patients. Three areas of focus are used: a) polypharmacy, b) cognitive issues such as delirium, dementia and depression, and c) increased odds of pathologies and chronic illnesses.

## Background

One of the greatest challenges in caring for older adult patients is formulating an accurate and appropriate diagnosis [1,2]. Older adult patients bring complex health histories and clinical scenarios into our practices, and it takes a skilled practitioner to overcome the related diagnostic challenges. These challenges have been discussed in the chiropractic and other health literature, often with an emphasis on improving patient care outcomes [2-5].

The fastest growing subset of the US population is the 75+ age category [6] and currently, about 15% of chiropractic patients are over 65. Most industrialized nations are seeing similar trends of rapid growth in the aging population. Many patients [in the US] seek chiropractic care for a chief complaint of musculoskeletal pain [7]. This is not surprising, considering that recent research shows that nearly one quarter of patients over the age of 75 experience non-disabling back pain, and another 6% report disabling back pain [8-11]. Chiropractors are well-suited to manage many patients with spinal complaints since chiropractic or spinal manipulative therapy is one of several efficacious evidence-based interventions for patients with back pain [12-19]. However, further research needs to be done to assess the efficacy and safety of chiropractic in the management of older back pain patients.

Due to this confluence of an aging world and the complex nature of geriatric health, it is essential that chiropractors thoroughly assess their older patients for concomitant health issues that may impact clinical decision making and patient prognosis. In this article a case-based approach will be taken to explore several important clinical problem solving examples. These cases are fictitious, based on an amalgam of previous clinical experiences, therefore no IRB or ethics approvals were required. The focus will be on three major confounding areas that create diagnostic challenges in managing older adult patients: Polypharmacy, cognitive issues, and increased risk of a co-morbid pathology or chronic illness.

## Main text

### Common age-related changes in older patients

In spite of the frequency in which chiropractic patients present with a musculoskeletal pain complaint, there is no doubt that common age related changes impact older patients profoundly. Such changes include [20]:

➢A decline in all five senses [vision, hearing, touch, taste and smell]

➢Lower muscle mass, bone density, and ligament elasticity

➢Some amount of atherosclerosis

➢Increased risk of co-morbid conditions [higher incidences of cancers, etc.]

➢Greater likelihood of chronic disease, like heart disease and arthritis,

➢Potential cognitive changes

It is with these changes in mind that examination of older patients should occur with a heightened vigilance for co-morbid conditions that may require medical care or concomitant care. One goal of a health professional in geriatric care should be to identify the diagnosis or to have a high index of suspicion of an important health concern.

#### Case: Sophie’s sniffle and slip

" Sophie, age 80, has a little cold. She takes an over the counter fizzy tablet [as she describes it] She reports that it helps with her cold symptoms. Unfortunately, last night Sophie got up to go to the bathroom and she got dizzy and fell. She comes to see you because she twisted her back and hurt her wrist in the fall. A careful examination strongly suggests that Sophie did not break any bones, and her aching back and wrist appear to be simple strains. The dizziness concerns you, so, you ask a local pharmacist if the cold medication she took the night before may have dizziness as a side effect. The pharmacist cautions that this particular medication can increase the risks of falls in older patients. She suggests several alternate over the counter medications that would be safer for Sophie. Subsequently, you may manage this patient with gentle chiropractic care, if indicated. "

## Medication issues

According to Healthy People 2020, polypharmacy is the principle drug safety issue in the US, particularly in older patients [21]. Polypharmacy is defined in a number of ways including: too many medications, inappropriate medications, bad combinations of medications, or medications being used to treat the side effects of other medications [21,22]. While prescription medication falls outside the scope of chiropractic practice, we do see patients who are taking many different medications. In fact, the average person over the age of 65 in America is taking 6–8 medications, often including several over the counter forms [23].

In the USA adverse drug events cause over 700,000 emergency department visits each year. Nearly 120,000 patients [all ages] each year need to be hospitalized for further treatment after emergency visits for adverse drug events. As more and more people take more medicines, the frequency of adverse events may increase. Older adults [65 years or older] are twice as likely as others to come to emergency departments for adverse drug events [over 177,000 emergency visits each year] and nearly seven times more likely to be hospitalized after an emergency visit [22,24,25]. Chiropractors must carefully note patient’s medications, and be vigilant for adverse events, which may even present simply as muscle or joint pain.

Several resources are available related to medication information [22,24-26]. Chiropractors may also want to consider teaming up with a local pharmacy to sponsor “Brown Bag Events”; when patients bring in all their medications in a brown bag, and take a list of medications to the local pharmacist to identify problems or concerns with their medications. If issues are detected, the patient [or pharmacist, or chiropractor] can request that their prescribing doctor conduct a medication review which may result in potential changes to the medication regimen.

## The 3 D’S: cognitive issues in the aging patient

### Delirium: the first of the 3Ds

Delirium or *acute* confusion is fairly common in older adult patients and may be frightening for both chiropractor and patient. Delirium is common and is a transient state, and nearly always resolves once the cause is determined and underlying issue is addressed [27]. Acute confusion may make it difficult to diagnose and care for the delirious chiropractic patient. Three major contributing factors in delirium stand out and each will be illustrated by a case below [27,28]:

#### Case: George’s bad day

" George, age 73, has been your patient for years, but today, he says it was the craziest thing. He got lost coming to your office, something he has done a hundred times. His brother, Bill, who is visiting, comes to George’s appointment with him, and is worried. He tells you in private that George has been acting strangely since he arrived, although he had been fine over the phone just a few days ago. George had a recent medical appointment, but now he seems “off”. He forgot to pick Bill up at the airport, seemed confused when Bill showed up at the house, seems to not know that his sister recently passed away, and has been “in a daze.” This sort of thing has never occurred before. George is usually as sharp as a tack. "

##### Discussion

this change in cognitive status prompts a focused examination and renewed history. George’s vital signs are all normal and he has not suffered any recent injury or emotional trauma. He does not drink alcohol and his diet has not changed. In further discussion with George, he reports that one of his anti-anxiety medications was changed a few days ago. With a quick call to the pharmacist about the new medication, the concern is raised that the new medication may be causing acute confusion [confusion is a common side effect of several common medications taken by older adults.]. A call is made to the prescribing doctor, and George is given the first available appointment. After a medical review and a change in the medications, George is back to his old self again in a few days and Bill is glad to have his brother back.

##### Medications

One of the most common causes of delirium is medications. Both over the counter medications and medications taken as prescribed may become toxic in older patients, resulting in confusion [27,28]. The only way to eliminate the delirium is to have the prescribing doctor or pharmacist identify and eliminate the medication or combination of medications which caused the confusion. Since many commonly used medications in older adults are taken for life-threatening diseases, medication evaluation should be overseen by a family medical doctor or a medical geriatrician/gerontologist and in consultation with a pharmacist where necessary.

#### Case: Hugh’s bump in the road

" You witness a minor car accident up ahead, so you stop to see if anyone was hurt. An older gentleman rear ended a man in his 30’s who appears to be very upset about the damage to his newer car. He approaches the older driver angrily as you peek into the driver’s window. Hugh, who looks to be in his 80’s is looking around nervously in the car and fumbling with the door. You help him to open the door and you see that he has a bad cut on his forehead. He’s bleeding and when you ask him if he is okay, he speaks unintelligibly, something about a horse and then says he has to get some milk. He gets out of the car with some unsteadiness then starts to walk out into the street, despite oncoming cars. When you try to stop him, he curses and takes a swing at you. "

##### Trauma

Acute injury may result in acute confusion. For example, a minor bump on the head may result in delirium, and create a confused and maybe even violent and unpredictable patient. It is essential to get immediate emergency treatment for an injured older adult, and to be aware that acute confusion will regress once the injury is resolved. Patients who present for chiropractic care due to a musculoskeletal injury may be difficult to properly assess due to delirium.

#### Case: Bill’s pain in the back

" Bill’s family is concerned. Bill, at age 75, is normally a mentally sharp man. Lately, however, Bill seems confused and has also been complaining of a “back ache” for 2 weeks. During your history and exam, you determine that he has had no change in medications, and no recent injury, however Bill’s back pain seems to be more than just musculoskeletal pain. He has an achy vague pain in his back, pelvic area and lower abdomen. Since he is over 50, with a high fat diet, and has a family history of prostate cancer, you know he is also at increased risk for prostate cancer. Even though you had encouraged him to have a baseline Prostate Specific Antigen [PSA] blood test and prostate exam several years ago, he has not had one for years. You ask the related history questions: “Do you have any changes in urination, defecation or sexual function?” His wife, who is in the room with Bill, chimes in, reporting that Bill has been getting up to urinate several times a night, which wakes her up. Bill confesses to having some burning and frequency of urination. Urinalysis reveals a bladder infection, so you inform Bill and his wife and send a copy of Bill’s urinalysis results to his doctor. The doctor requests to see Bill that day, and Bill’s wife takes him in. One day after Bill starts taking his antibiotics, his urinary frequency and pain is resolved, along with the supra-pubic pain. His wife calls to tell you his confusion has completely gone away. "

##### Infection

A simple bladder infection or respiratory infection may have a unique presentation in elderly patients. In fact, all other symptoms may be eclipsed by the erratic, confused behavior. In the case above Bill had a simple bladder infection, secondary to benign prostatic hypertrophy [BPH]. It is important to note that an estimated 50% of men demonstrate histopathologic BPH by age 60 years. This number increases to 90% by age 85 years [29]. Missing this diagnosis may result in delirium that may not resolve until the infection has been resolved. Long term confusion may be mistaken for dementia, one example of a very serious misdiagnosis.

### Dementia: the 2^nd^ of the 3Ds

Dementia is a collection of symptoms including slow, gradual onset memory loss, intellectual impairment, loss of emotional control and problem solving ability. One type, Alzheimer’s Dementia [AD] leads to a progressive loss of mental functions, and often includes disruption of speech or difficulty finding words [30-32]. It is estimated that 5 million people are currently living in the US with AD, and about 360,000 people are diagnosed each year with over 50,000 death attributed to AD annually [32]. The case below may present to chiropractors showing a gradual onset of several of the main symptoms of AD. In recent years, research has shown that heart health and brain health are related, and patients may slow the progression of AD by making heart healthy lifestyle choices [31,32]. Chiropractors should consider assessing confused patients with the Mini-Mental Status Exam [MMSE] and discussing the patient’s score with his or her medical provider [33].

#### Case: Henry is lost

" Henry, age78, has been your patient for a decade, but you have noticed his mental sharpness has been in decline over the past few years. He remarks that he got lost coming to your office today, and he confesses that he got lost going to the grocery store twice in the past 2 months. As he speaks, he verbally wanders and seems to have difficulty finding the words to complete his sentences. He says he has good and bad days, but lately there have been more and more bad days. Henry becomes emotional as he tells you for the second time about getting lost today. He is noticeably shaken up. Henry has a history of cardiovascular health issues and does little, if any, physical activity. "

## Discussion

While a chiropractor can potentially treat Henry for his musculo-skeletal problems, medical oversight is required to further assess his dementia. It is essential that his cardiac health be monitored, so it is important for the chiropractor to regularly take blood pressure and to make certain that Henry is regularly consulting his medical physician. Dementia can be devastating for both patient and family, so it is important that the family identify resources related to dementia such as the Alzheimer’s Association (USA) [34].

### Depression: the 3^rd^ of the 3Ds

Depression is a common mental health concern in older adults. One in 5–10 elderly adults suffer from depression and 80% of all depression is completely treatable, once detected. Detection is of utmost importance since suicide is one of the top 10 causes of death in older patients [35]. Suicide among white males aged 85 and older was nearly six times the national U.S. rate, making this a serious concern for all health professionals. Older adults often have significant risk factors for depression such as chronic illness and/or chronic pain. Poor diet and poor absorption of B vitamins in the aging body, may also contribute to depression [28,35]. Older adults are often faced with the death of a spouse, friends or peers. Significant life events such as these, combined with all the factors above, create fertile ground for depression to take hold in the elderly patient. Chiropractors may see depressed older patients since depression often magnifies symptoms of musculoskeletal pain [28,35].

It is important that all health professionals caring for the older adults be aware of the diagnostic criteria for depression [36]. Further, if a patient reports being sad or depressed it is important to assess the patient more thoroughly using a Geriatric Depression Scale [37] or similar assessment tool, which takes only a minute or two for patients to complete. Online versions can readily be downloaded. Scores on such forms should be discussed with their treating medical doctor, a psychologist or psychiatrist to determine an appropriate and safe course of care, and improve patient outcomes.

## Common co-morbidities and pathologies

### The heart of the matter

According to World Health Organization statistics, heart disease is the number one cause of death worldwide, accounting for 7.2 million deaths annually [38]. Cerebrovascular disease [a related disorder] is the second leading cause of death with another 3 million deaths annually. In Australia, heart disease is also the leading cause of death with nearly 50,000 deaths annually [39]. Using America as another example, heart disease is the top killer of both men and women, with most deaths in patients over age 60 [40]. About 18 million Americans currently have heart disease, and about half of those are over age 60 [41]. The 2011 published statistics from the American Heart Association, state that from 1997 to 2007, the total number of inpatient cardiovascular operations and procedures increased by 27 percent. The estimated total cost from heart disease and stroke in the United States for 2007 [including health expenditures and lost productivity] was $286 billion, which is higher than for any other diagnosis [40].

The case below will illustrate why heart disease should be on the mind of chiropractors every day in practice. Nearly all chiropractors, if they are in practice long enough will have a patient present for chiropractic care while they are having a heart attack. It is essential that chiropractors be good diagnosticians, so as to avoid a delay in emergency care for patients experiencing a cardiac event.

#### Inez’s bad day

" Inez is 65 years old. She cares for 3 of her grandchildren at home on a regular basis, getting two of them ready for school, and one to day care each morning. One morning, in the midst of her busy routine, Inez started feeling queasy and uncomfortable. By the time she dropped the 3^*rd*^child off at daycare, Inez also started having neck and jaw pain. She headed straight to your chiropractic clinic, as she has been a patient there before for her occasional neck pain. Today, you examine her and decide to take her blood pressure and pulse since her blood pressure has increased over the past few years and she reported last year that her doctor had mentioned that she may need to go on medication. She is not currently taking any anti-hypertensives, but is taking oral medications for type 2 diabetes. Her blood pressure was high [150/100] and her pulse was fast. In evaluating her neck and jaw, movement did not make it worse. You ask her to march in place with high steps for 1 minute to see if exertion makes it worse, and sure enough, she has to stop due to “feeling sick”. You call for emergency services and arrange an ambulance. Inez was having a heart attack, and she calls you once she is out of the hospital to thank you for “saving her life.” "

##### Discussion

Heart attacks in women don’t always follow the rules. Heart attacks, after all, are supposed to present as crushing chest pain, and left arm pain. But, female heart attacks often result in vague pain, no pain, discomfort or just an uneasy feeling [39]. While awareness among doctors is improving, women used to have twice the chance of dying from a heart attack than a man, in part due to missed diagnoses in the emergency room [27,39]. However, since the symptoms are so subtle and unpredictable, many women fail to go to the hospital during a heart attack, making treatment impossible. It is important that chiropractors be aware of the way cardiac events mimic musculoskeletal pain in many cases.

## Notes on care of the older patient with back pain

Among older adults, back pain is one of the leading causes of a trip to the doctor [8-10]. It is essential that a chiropractor know some of the factors that make back pain more challenging in older adults. Older adults often have stiffness as a chief complaint, along with limits in their range of motion and functional ability. For many older patients, the most worrying factor about musculoskeletal pain is that it might rob them of their independence. Not being able to turn one’s neck may translate into “having to give up driving.” Not being able to bend, may translate into, “not being able to pick up the grandkids.” Not being able to stand up from a seated position comfortably, may translate to “needing help in the bathroom.” As you can see, functional outcomes may be more important to the older adult than any other patient age group.

It is beyond the scope of this article to point out the specifics of care and assessment of patients with back pain, but excellent literature has been presented elsewhere including in this journal’s series on the elderly.

## Increased risk of pathology

For many younger patients, musculoskeletal pain may be a simple as a sprain or strain, including the common presentation of uncomplicated back pain [7,13-15]. In fact, a high percentage of the cases chiropractors see [in all ages of patients], it is likely that a simple course of chiropractic care may address the problem and the patient’s functional status and pain will improve [11,13,14]. While older patients also may enjoy good results with chiropractic care, the odds of complicating factors and comorbidities are much higher. Some examples of co-morbidities that complicate the care of older adults are [38-44]:

1. Arthritic changes

2. Spinal stenosis [narrowing of the vertebral canal]

3. Osteoporosis with possible compression fractures

4. Pathologies [metastasis of cancer from other sites, other bone tumors]

5. Organ dysfunction causing back pain symptoms [prostate, heart, lung]

The table below indicates some of the incidences of common health concerns in older adults using the United States as an example [40-43]:

All of these disease processes are more likely in the older adult [38-45]. These statistics help emphasize the need for diagnostic imaging of the older patient when the history, examination, and symptoms indicate something beyond uncomplicated back pain.

The odds of pathology are higher in the older patient than in any other age group. Sixty per cent of the metastases to the spine are from the prostate [almost exclusively in patients over 50] and another 25% of metastases are from the lung [also more common in older patients with a history of smoking.] Breast cancer metastases to the spine are also predominantly seen in older patients [43,45].

Recent imaging prior to initiating manual therapy for older patients may be useful to avoid damaging anatomical structures that contain underlying pathology. Although the definition of “older” in the case of routine imaging is controversial, with some now recommending 65 and older as the age of concern for low back pain [46]. Incidental findings, such as osteoporosis, degenerative joint disease, hiatal hernias, abdominal aortic aneurisms, stenosis, and so on, are all more likely in the older patient. Some of these findings may indicate the need for medical co-management, while others may influence the chiropractor’s choice of management strategies.

## Discussion and conclusion

In this article we have explored just a few cases that highlight the diagnostic challenges in the older adult patient. As the baby boomers reach their golden years, it is essential that all health professionals learn more about normal aging, and common health related concerns in older patients. Excellent resources for providers are available at the National Institute on Aging at http://www.nia.nih.gov[47] and the American Geriatric Society http://www.healthinaging.org/agingintheknow/[48] These and other resources can be utilized to stay abreast of the latest health information related to caring for the older adult patient.

## Competing interest

The author declares no competing interests or financial interests.
